# Identification of a chimeric e*mm* gene and novel e*mm* pattern in currently circulating strains of e*mm4* Group A *Streptococcus*

**DOI:** 10.1099/mgen.0.000235

**Published:** 2018-11-09

**Authors:** Sruti DebRoy, Xiqi Li, Awdhesh Kalia, Jessica Galloway-Pena, Brittany J. Shah, Vance G. Fowler, Anthony R. Flores, Samuel A. Shelburne

**Affiliations:** ^1^​ Department of Infectious Diseases Infection Control and Employee Health, University of Texas MD Anderson Cancer Center, Houston, TX, USA; ^2^​ Graduate Program in Diagnostic Genetics, School of Health Professions, University of Texas MD Anderson Cancer Center, Houston, TX, USA; ^3^​ Department of Genomic Medicine, University of Texas MD Anderson Cancer Center, Houston, TX, USA; ^4^​ Division of Infectious Diseases, Department of Pediatrics, University of Texas Health Science Center McGovern Medical School, Houston, TX, USA; ^5^​ Division of Infectious Diseases, Duke University Medical Center, Durham, NC, USA; ^6^​ Center for Antimicrobial Resistance and Microbial Genomics, University of Texas Health Science Center, McGovern Medical School, Houston, TX, USA

**Keywords:** group A *Streptococcus*, *emm*/*enn* gene fusion event, chimeric M protein, *emm* pattern

## Abstract

Group A *Streptococcus* (GAS) is classified on the basis of the sequence of the gene encoding the M protein (*emm*) and the patterns into which *emm* types are grouped. We discovered a novel *emm* pattern in *emm4* GAS, historically considered pattern E, arising from a fusion event between *emm* and the adjacent *enn* gene. We identified the *emm–enn* fusion event in 51 out of 52 *emm4* GAS strains isolated by national surveillance in 2015. GAS isolates with an *emm–enn* fusion event completely replaced pattern E *emm4* strains over a 4-year span in Houston (2013–2017). The novel *emm–enn* gene fusion and new *emm* pattern has potential vaccine implications.

## Data Summary

1. The Geneious software is available for purchase at https://www.geneious.com/.

2. The programs used to analyze single-nucleotide polymorphisms (SNP) and reconstruct whole-genome-sequencing-based phylogenies are publicly available at (a) kSNP v3.0 - https://omictools.com/ksnp-tool, (b) parsnp v1.2 - http://harvest.readthedocs.io/en/latest/content/parsnp.html and (c) FastTree 2 - http://www.microbesonline.org/fasttree/.

3. BioProject numbers for all reference genomes as well as the CDC collection of genomes used in the analyses are listed in the Data Bibliography section.

4. The short-read sequences associated with Chalker *et al*. can be found in the European Nucleotide Archive and are listed in the Data Bibliography section.

**Impact Statement**

Group A streptococci (GAS) are a major cause of serious infections in humans, ranging from simple pharyngitis (strep throat) to life-threatening diseases, such as necrotizing fasciitis (the flesh-eating disease). One of the critical virulence factors of GAS is the hypervariable M protein, which is the primary target for the human immune system and a leading vaccine candidate. GAS strains are well known to slightly vary the sequence of the *emm* gene and hence the M protein in order to establish infections in humans. In this study, we characterize a novel *emm* gene which has resulted from fusion of the existing *emm* with its neighboring *enn* gene, theoretically resulting in a completely new M protein rather than the minor variations previously described. We found that GAS strains causing both invasive and superficial infections that carry this new *emm* gene have been replacing strains with the old *emm* version. Collectively, the outcomes of this study provide a framework to further investigate the contribution of the new *emm* gene to the emergent dominance of the new *emm* genotype in human populations.

## Introduction

**Fig. 1.**
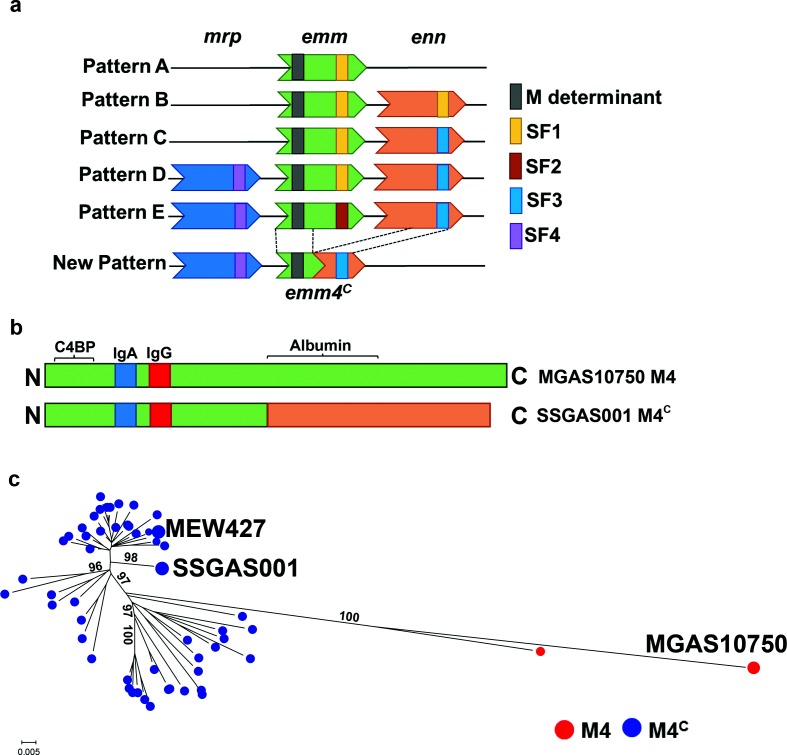
Characterization of GAS isolates. (a) Schematic diagram to compare the new *emm* pattern with existing patterns (adapted from reference [3]). The *emm* type-specific (M) determinant and the subfamily (SF) of the PG domain is indicated. (b) Diagram showing predicted binding sites for IgA, IgG, C4BP and albumin based on motifs [1] present in the deduced sequence of the M protein from MGAS10750 and SSGAS001. (c) Maximum-likelihood tree reconstructed using 814 core SNPs of *emm*4 GAS strains from CDC [11] along with publicly available *emm4* GAS genomes (names are provided). The local support values are provided. The type of M protein is color coded as indicated in legend.

Group A *Streptococcus* (GAS) is among the most ubiquitous human pathogens and is classified into *emm* types based on the 5′ sequence of the *emm* gene that encodes the hypervariable M protein, a key GAS virulence determinant [1]. GAS infection is thought to engender serotype-specific immunity, and the M protein or its domains are leading vaccine candidates [2]. In addition to invariably containing the *emm* gene, the *emm* region can also contain one or two *emm*-like genes, typically designated as *mrp* and *enn* (Fig. 1a) [3]. The 3′ end of the *emm* and *emm*-like genes encode a peptidoglycan–spanning (PG) domain, and PG sequence variation determines four subfamilies (SF-1 through SF-4, Fig. 1a). The composition of *emm*-family genes along with their respective PG domain subfamily types divides GAS strains into five *emm* patterns (A–E), and for the overwhelming majority of GAS strains studied to date, there is strict concordance between *emm* type and *emm* pattern [3]. The *emm* pattern of GAS strains strongly correlates with the preferred epithelial site of infection i.e. pharynx versus skin [3]. Thus, *emm* pattern A–C strains are considered ‘throat specialists’, whereas pattern D are ‘skin specialists’, and pattern E are ‘generalists’ [4].

The majority of data establishing the five *emm* patterns and their relationship to *emm* type were generated from strains isolated in the 1980s and 1990s using targeted approaches like PCR and Southern blotting [4, 5]. Initial whole-genome sequencing (WGS) of one or a few GAS strains per *emm* type done in the 2000s supported these findings [6]. However, the past decade has witnessed a marked increase in WGS of large cohorts of GAS strains, which has revealed significant intra-*emm* type diversity [7]. Moreover, the highly antigenic and thus hypervariable nature of the M protein has long been recognized [8]. Therefore, with the additional density of sequencing data and the elapsed time since the recognition of the five *emm* patterns, one might predict that additional variations in the *emm* region would be recognized. Herein, we report the identification of genetic event in currently circulating *emm4* strains, previously considered pattern E, that give rise to a chimeric *emm* gene and a novel GAS *emm* pattern.

## Methods

The invasive GAS *emm4* strain SSGAS001 was isolated from a point-source outbreak in 2015 and its genome described using the strain name ‘Duke B’ [9]. We accessed the complete genomes of the 2001 pharyngeal isolate MGAS10750, considered the *emm4* reference strain, and the 2015 pharyngeal *emm4* isolate MEW427 from NCBI [6, 10]. Short-read sequencing data of invasive *emm4* strains collected by the Centers for Disease Control and Prevention (CDC) during 2015 [11] and in England in 2014 [12] were accessed from BioProject PRJNA395240 and the European Nucleotide Archive project ERP015112 respectively. *Emm* region sequences were extracted from annotated draft genomes and visualized through Geneious (Biomatters). Two core single-nucleotide polymorphism (SNP)-based methods were deployed to reconstruct WGS-based phylogenies: (a) kSNP v3.0 [13] identified core SNPs based on reference free k-mer analysis (k-mer size of 19, default settings). These SNPs were used to reconstruct a maximum-parsimony tree; and (b) parsnp v1.2, available in the Harvest suite [14], was used to align assembled *emm4* genomes to the MGAS10750 reference, and SNPs identified in local collinear blocks were subsequently used for reconstructing an approximate maximum-likelihood tree using FastTree 2 [15] while incorporating the general time reversible (GTR) model of nucleotide substitution. The Shimodaira–Hasegawa test implemented in FastTree2 was used to assess the support for significant clustering in the observed phylogeny. Due to similar findings, only trees reconstructed by parsnp are provided. A total of 88 *emm*4 GAS were identified among 930 GAS isolates collected at Texas Children’s Hospital in Houston (Texas, USA) between 2013 and 2017 [16] under a protocol approved by the Institutional Review Board at Baylor College of Medicine. The *emm* region was amplified by PCR and subject to Sanger sequencing (primer sequences in Table S1, available in the online version of this article). Taqman qRT-PCR was performed as described previously [17]

## Results

*Emm4* strains commonly cause both invasive and non-invasive GAS disease and are considered a prototype *emm* pattern E GAS [1, 18]. We previously compared the complete genome of an invasive *emm4* strain isolated in 2015 (strain SSGAS001) to that of the reference *emm4* strain MGAS10750, which was isolated in 2001 [6, 9]. Closer examination revealed a fusion event between *emm* and the immediately downstream *enn* gene in SSGAS001 resulting in a chimeric *emm* gene, which we have designated as *emm4^C^*. The *emm4^C^* gene is a fusion of the 5′ end of the *emm* and the 3′ end of the *enn* gene of MGAS10750 (Fig. 1a). Given that the 5′ of *emm4^C^* is unchanged, SSGAS001 is still categorized as *emm4*. However, the C-terminal half of the *emm4^C^*-encoded protein, M4^C^, only shares 62 % identity with the C-terminus of the MGAS10750 M4 protein. Moreover, the chimeric M4^C^ protein contains an SF-3 allele in the PG domain rather than the SF-2 allele found in the PG domain of the MGAS10750 M protein. Given that the majority of M protein function has been mapped to the N-terminal region [1], the M4^C^ protein is not predicted to have alterations in binding IgA, IgG, or C4BP, but interaction with albumin could be affected (Fig. 1b). In addition to generating a new predicted chimeric M protein, the *emm–enn* gene fusion event observed in SSGAS001 also results in just two genes in the *emm* region (*mrp* and *emm*) instead of the three (*mrp*, *emm* and *enn*) that are characteristic of Pattern E GAS (Fig. 1a). Thus, the gene fusion event gives rise to an *emm* pattern heretofore undescribed in GAS strains. We analyzed the other fully sequenced *emm4* GAS strain, MEW427, also isolated in 2015 [10], and found it to have the identical *emm4^C^* gene and *emm* pattern to strain SSGAS001.

Although study of *enn* is limited, there have been reports of low *enn* transcription and/or translation, perhaps due to an unusual start codon [19–22]. We found that *enn4* transcript level was approximately 20-fold lower than that of *emm4* in MGAS10750 (Fig. S1) and that *enn4* in MGAS10750 is predicted to have a non-standard start codon (isoleucine rather than methionine). Thus, Enn in MGAS10750 is likely to be produced at low levels.

**Fig. 2.**
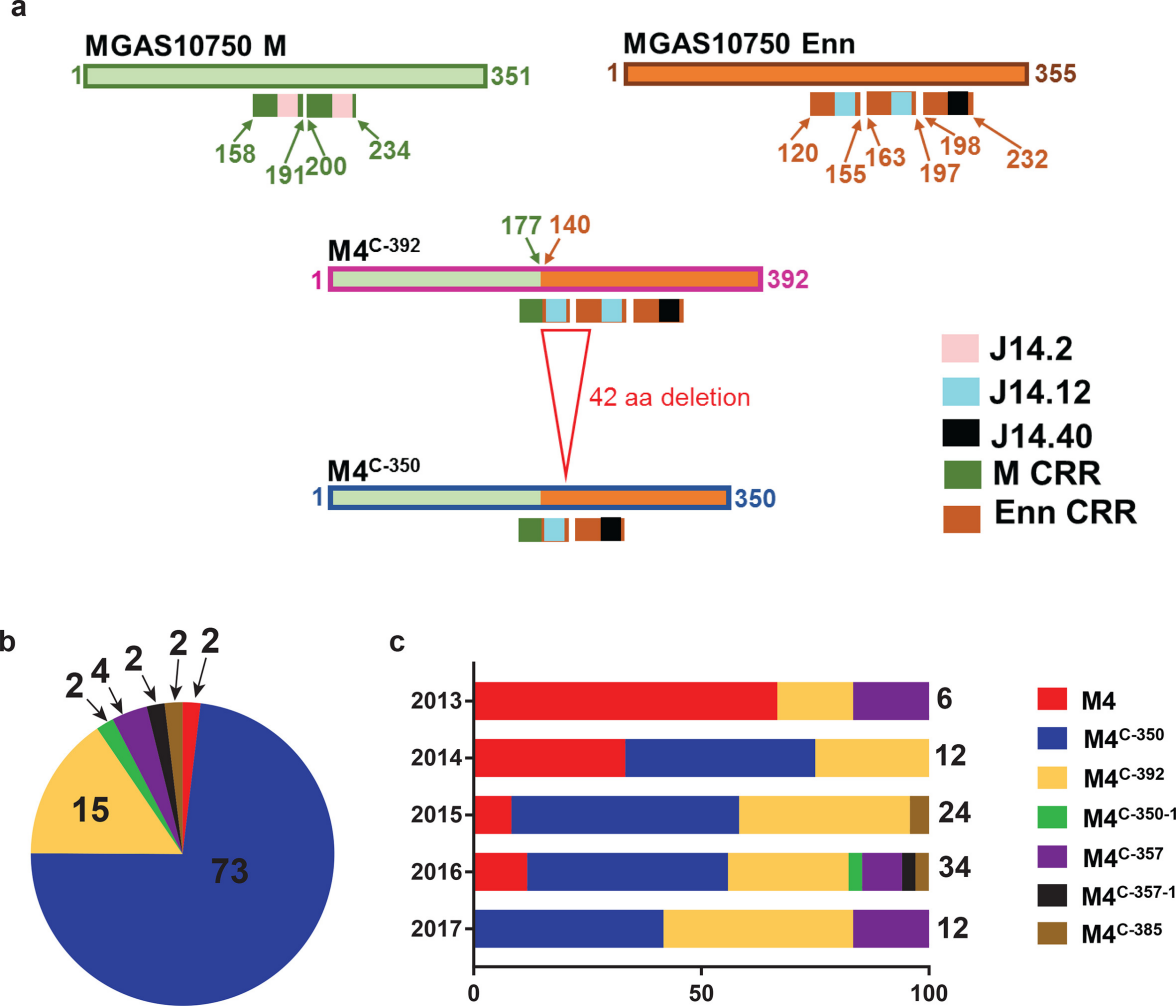
(a) Schematic diagram showing the two most common chimeric M protein variants. The fusion point of the *emm* and *enn* genes is indicated by the change in box shading with numbers indicating the last amino acid from *emm* and the first amino acid from *enn* respectively in M4^C^ protein. The 42 amino acid (126 nucleotide) deletion that is the difference between M4^C-392^ and M4^C-350^ is noted. C-terminal repeat regions (CRRs) along with J14 sequences are displayed as color-coded boxes. (b) Percentage distribution of the canonical and chimeric M4 proteins in 52 *emm*4 GAS strains collected by the CDC in the USA in 2015 depicted as a pie-chart. Numbers indicate percentages of the total. (c) Distribution of the canonical M4 and the different M4^C^ variants identified in 88 *emm4* strains isolated from patients at Texas Children’s Hospital between 2013 and 2017 expressed as a percentage of the number of isolates per year. Total number of isolates in a given year is indicated on the right of the bars. There are relatively fewer isolates in 2013 because collection only began in May, 2013. For (b) and (c), the type of M protein is color coded as indicated in the legend.

To determine the frequency of the gene fusion event amongst currently circulating *emm4* GAS strains, we first analyzed published WGS data from 52 invasive *emm4* isolates collected by the CDC in 2015 [11]. We identified the *emm–enn* gene fusion event in 51 out of 52 strains with only a single strain containing *emm* pattern E (Fig. 2b, Table S2). Whole-genome-based phylogeny revealed that strains with the chimeric *emm4^C^* gene clustered distinctly from strains with the canonical *emm4* gene (Fig. 1c). Importantly, the chimeric *emm4^C^* genes are present in strains isolated from multiple locations in the USA rather than being confined to a single location (Fig. S2).

We identified six variations of the predicted M4^C^ protein (Figs 2a and S3). The 350 amino acid long variant, which we have named M4^C-350^, occurred most commonly and was identical to M4^C^ in strains SSGAS001 and MEW427. M4^C-350^ differs from the other common M4^C^ variant, M4^C-392^, by a 126 nucleotide (42 amino acid) deletion (Fig. 2a). Together M4^C-350^ and M4^C-392^ are present in 90 % of strains in the CDC dataset that have the *emm–enn* gene fusion event (Fig. 2b). The other four variants appear to be derived via insertions or deletions in the M4^C-392^ or M4^C-350^ protein (Fig. S3). To determine if the presence of the chimeric *emm–enn* fusion was limited to strains in the USA, we analyzed short-read data for 33 *emm4* isolates collected in England in 2014 [12]. We detected the *emm–enn* fusion event in 84 % of these strains.

Although the N-termini of the predicted M4^C^ proteins are identical to the canonical M4 protein, the M4^C^ proteins have different C-terminal repeat regions (CRRs). The CRRs represent a domain within the cell surface-exposed portion of the M and Enn proteins that contain tandemly arranged blocks of direct sequence repeats which have been targeted as potential GAS vaccine candidates [23]. The sequence repeats are not always identical and can be further classified based on the sequence of the C-terminal 14 amino acids (J14) in a given repeat [24]. The MGAS10750 M4 protein contains two CRRs, both containing the J14.2 sequence. Unlike MGAS10750, M4^C-392^ contains three CRRs, but other M4^C^ variants also possess two CRRs each. However, the composition of the CRRs in every M4^C^ variant differs from that in MGAS10750 in terms of the J14 sequence (Figs 2a and S3, Table S2).

To assess temporal emergence of *emm4* strains with a chimeric *emm4^C^* gene, we analyzed 88 invasive and non-invasive *emm4* isolates collected at the Texas Children’s Hospital in Houston between 2013 and 2017 via Sanger sequencing of the *emm* region (Table S3) [16]. We identified the *emm–enn* gene fusion in 74 strains (84 %). While 66 % of the *emm4* strains isolated in 2013 were still the canonical *emm* pattern E, there was total replacement by strains carrying *emm4^C^* by 2017 (Fig. 2c). Similar to the CDC strains, the M4^C-350^ and M4^C-392^ variants were most common among the Houston isolates. Consistent with the hypothesis that M4^C-350^ arose from M4^C-392^, M4^C-350^-containing strains were not detected until one year after the initial identification of strains with the predicted M4^C-392^ protein (Fig. 2c).

## Discussion

Since its discovery some 90 years ago, M protein has been considered the key GAS virulence determinant and is currently a prime vaccine candidate [2]. Despite extensive investigations over the past decades, continued study of M protein and the *emm* region continues to uncover new findings. Herein, we demonstrate that over 80 % of recently circulating *emm4* GAS strains in the USA and England contain a non-canonical chimeric *emm4^C^* gene and a previously unrecognized *emm* pattern.

The key finding of this study was identification of a gene fusion event that gave rise to multiple novel entities. First, it created a chimeric *emm* gene, *emm4^C^*, that consists of the 5′ end of the reference *emm4* gene and the 3′ end of the *enn4* gene. Second, formation of the chimeric *emm4^C^* gene eliminated the *enn* gene, thereby producing an *emm* region that only contains *mrp* and *emm*, a pattern different from the five established ones. Finally, unlike previously reported M proteins, which harbor either a SF-1 or SF-2 allele in the PG domain [3], the predicted M4^C^ carries the SF-3 allele of the PG subfamily.

Analysis of 173 recent *emm*4 isolates revealed that strains with the chimeric *emm4^C^* gene and the new *emm* pattern have almost completely replaced the previously circulating *emm4* pattern E strains in the USA and England. This indicates that the *emm–enn* gene fusion may confer a selectively advantageous trait, by itself or together with additional genomic changes in *emm4^C^* strains, which drove the emergent dominance of the *emm4^C^* strains in the studied populations. Given the critical nature of M protein in GAS host–pathogen interaction, it is plausible that the gene fusion event is at least a partial catalyst for the observed replacement.

M protein sequence variation can confer changes in immune recognition and has been associated with clonal population expansion [7]. However, reports of M protein variation have described small-scale changes (i.e. insertions, deletions, tandem repeat variation) in the N-terminal region whereas we observed replacement of the entire C-terminus [7, 8]. Although the N-terminus is typically considered to be the portion of the M protein under selective immune pressure [1], there are also data supporting immunogenicity of the C-terminal region. Like other M4 strains, Enn in MGAS10750 contains an unusual start codon and low *enn* transcript level, indicating that Enn4 may not have engendered an immune response in older M4 strains, consistent with reports from other serotypes [20, 25]. Hence, these data indicate that the *emm–enn* gene fusion may have significant immunological effects on GAS–host interactions, but this needs to be experimentally tested.

In summary, we report the occurrence of a recombination event that has given rise to a chimeric *emm* gene and a novel *emm* pattern and provide evidence of its predominance among current *emm4* strains. These data indicate that GAS mechanisms that alter the M protein are more varied than previously appreciated, which could affect the efficacy of M-protein-based vaccine strategies.

## Data bibliography

1. Galloway-Pena J, Clement ME, Sharma Kuinkel BK, Ruffin F, Flores AR, *et al*. NCBI BioProject accession #PRJNA300859.
2. Beres SB, Richter EW, Nagiec MJ, Sumby P, Porcella SF, *et al*. NCBI BioProject accession #PRJNA224116.
3. Jacob KM, Spilker T, LiPuma JJ, Dawid SR, Watson ME, Jr. BioProject accession# PRJNA308988.
4. Chochua S, Metcalf BJ, Li Z, Rivers J, Mathis S, *et al*. Population and Whole Genome Sequence Based Characterization of Invasive Group A Streptococci Recovered in the United States during 2015. BioProject accession# PRJNA395240.
5. Chalker V, Jironkin A, Coelho J, Al-Shahib A, Platt S *et al*. Genome analysis following a national increase in Scarlet Fever in England 2014. European Nucleotide Archive project number ERP015112.
6. The Illumina short read data for SSGAS001 is available as BioProject # PRJNA300859 with the strain name of SASM4-Duke.
7. The complete and annotated genome of SSGAS001 can be found in Genbank under the accession number CP031770.

## Supplementary Data

Supplementary File 1

Supplementary File 2
