# Supplementary material for: Identification of a chimeric emm gene and novel emm pattern in currently circulating strains of emm4 Group A Streptococcus
Source: Microb Genom. 2018 Nov 9;4(11):e000235. doi: 10.1099/mgen.0.000235 (PMC6321872; doi:10.1099/mgen.0.000235)
Supplement: Supplementary File 1 [file mgen-4-235-s001.pdf]

Supplemental Data

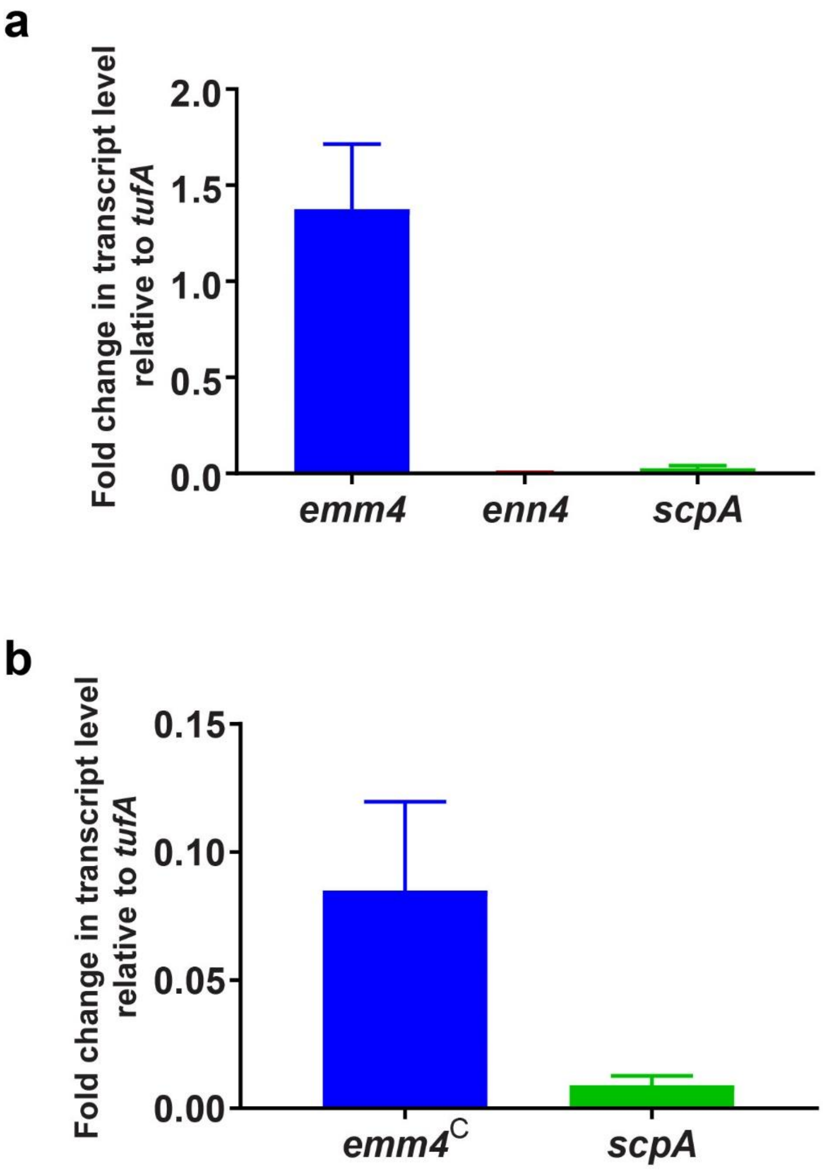

Supplemental Figure 1. Taqman qRT PCR analysis to verify the transcript levels of *emm*, *enn* and *scpA* (encoding C5a peptidase) in (a) MGAS10750 and in (b) SSGAS001. Data represents the mean ± standard deviation of strains grown in duplicate on two separate days and analyzed in duplicate (total of 8 data points). Due to the chimeric nature of *emm4*^{C}, different primer/probe sets were used to detect the *emm* transcript from

MGAS10750 and SSGAS001 and thus a direct comparision between *emm4* and *emm4*^{C} cannot be made.

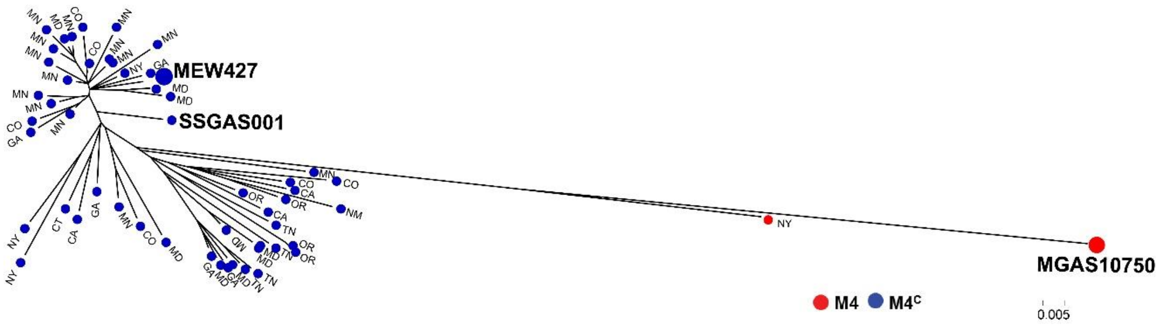

Supplemental Figure 2. Maximum-likelihood tree reconstructed on 814 core SNPs of *emm*4 GAS strains from CDC [1] along with publicly available *emm4* GAS genomes (names are provided). The state abbreviations next to each strain notes location of isolation.

1. Chochua S, Metcalf BJ, Li Z, et al. Population and Whole Genome Sequence Based Characterization of Invasive Group A Streptococci Recovered in the United States during 2015. MBio **2017**; 8.

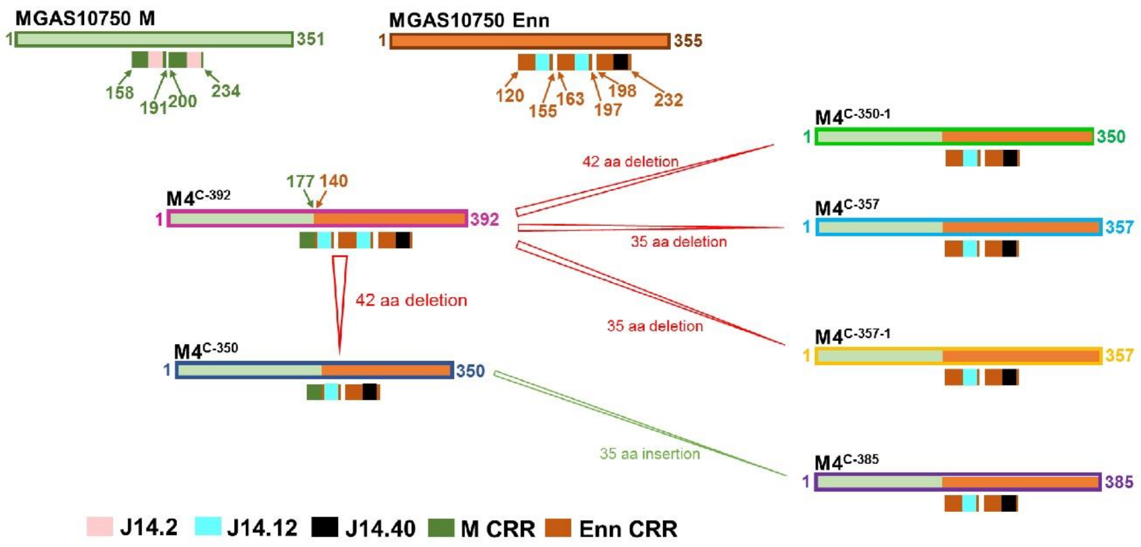

Supplemental Figure 3. Schematic diagram showing all six different predicted chimeric M protein variants. Arrows indicate putative deletion or insertion events that lead to the formation of each of the M protein variant. Fusion points are indicated (only for $M4^{C-392}$) by color-coded lines and C-terminal repeat regions (CRRs) are displayed as color-coded boxes.
